# Diseases of the Circulatory System

**Published:** 1901-09-28

**Authors:** 


### Diseases of the Circulatory System.

**Persistence of the Ductus Arteriosus.**—Gibson[1] summarises as follows the essential facts on which the diagnosis of a persistent ductus arteriosus may be confidently made. There may be no dyspnœa, cyanosis, œdema, or other evidence of disturbance of the general circulation. Palpation usually reveals a long thrill following the apical impulse and enduring beyond the recoil of the blood on the semilunar cusps which may be felt during the thrill. Auscultation gives evidence of the lesion in a murmur which may be regarded as almost pathognomic. Beginning directly after the first sound, it accompanies the latter part of that sound, occupies the short pause, accompanies the second sound, which may be accentuated in the pulmonary area or may be doubled, and finally dies away during the long pause.

**Apical Presystolic Murmurs.**—Gibbes[2] has usefully summarised the various conditions other than mitral stenosis which may occasion an apical presystolic murmur. It used to be thought that such a murmur was pathognomic of mitral stenosis, but such a view has lost ground since Flint drew attention to its occurrence in cases of aortic regurgitation where no stenosis of the mitral orifice was found on post-mortem examination. The presystolic murmur has been proved to be present under the following circumstances:—1. Obstruction of the mitral orifice by sclerosis and adhesion of the cusps or by invagination of an auricular thrombus. 2. Dilatation of the mitral orifice with normal cusps and chordæ. 3. The mitral orifice of normal size but toughened margins. 4. Insufficiency of the aortic valves, with (*a*) normal mitral orifice and curtains; (*b*) dilated mitral orifice but normal cusps and chordæ; and (*c*) mitral orifice dilated and cusps and chordæ thickened. We also find that it is equally present when the regurgitant current is directed towards the anterior mitral cusp, and when it is directed towards the septum, when it is free and when it is limited, and when there is no aortic or mitral regurgitation at all, the only morbid condition common to all being dilatation and hypertrophy of the heart.

**Mitral Stenosis.**—We owe to Walsham[3] a most conclusive proof of the old opinion that the murmur of mitral stenosis is presystolic and not systolic in time. It will be remembered that some observers, *e.g.*, Dickinson, have urged that a mitral stenosis is really systolic in time. The proof is by the application of the X-rays, and anyone who has the apparatus can satisfy himself of its truth. If the normal heart be observed on the fluorescent screen the movement of the apex can be seen to be a rapid one toward the left, followed by a retreat more rapid still towards the middle line. It is not the apex, however, but the middle of the left border which presents the greatest displacement. If the heart be listened to with a binaural stethoscope it is found that the first sound of the heart corresponds to the exact moment when the shadow begins to retreat towards the middle line. The projection of the shadow to the left is therefore presystolic and corresponds to the systole of the auricles, whilst the movement of retreat corresponds to the systole of the ventricles. The visible impulse is a presystolic phenomenon due to the systole of the auricles and to the consecutive distension of the ventricles. Now, if a well-marked case of pure mitral stenosis be taken, and the movements of the heart watched on the screen, and at the same time (as can be easily done) the apex beat be felt, it is found that the thrill corresponds to the advance of the left border of the shadow of the heart to the left and ends abruptly with the retreat of the shadow towards the middle line; or if the heart be listened to it is found that the stenosis murmur has the same time relations. It is therefore certain that both thrill and murmur are presystolic in time, taking place during the contraction of the auricles.

**The Heart Reflex.**—Abrams[4] has recorded some valuable observations which probably go far to explain the good results of Schott's treatment of heart disease. The reflex is seen by means of the Roentgen rays. If a fluorescent screen be placed close to the chest wall, and the skin of the precordial region be vigorously rubbed, for instance, with a piece of rubber, a contraction of the myocardium is observed, as a rule more manifest in the left than the right ventricle. The duration of the contraction is not less, as a rule, than two minutes, and it continues after the source of cutaneous irritation is removed. The degree of contraction, as observed by the diminution in the size of the shadow thrown on the screen, varies greatly. In some persons it is scarcely perceptible, whilst in other individuals the heart may recede fully an inch on either side. The nearer we approximate the precordial region and the more vigorous the cutaneous friction, the more pronounced is the cardiac reflex. In individuals with dilated hearts the reflex is very great and of much longer duration than in healthy hearts. Cutaneous stimulation of the chest, however, not only produces a cardiac reflex, but also a pulmonary one. If the skin of the precordial area, for instance, be vigorously rubbed, the area of superficial cardiac dulness is obliterated; or if irritation of the skin over the lower right intercostal spaces be made, it can be found by percussion that the lower border of the lung descends as much as two or more inches. The same phenomenon can be seen by the fluoroscope; coincident with the discharge of the reflex the lung area implicated shows increased brightness lasting from a few seconds to two minutes. If, then, the precordial region be rubbed in a case of dilated heart, and the area of dulness be marked out before the rubbing, just afterwards, and again in about three minutes after the rubbing, it is found that the area of dulness contracts a great deal (due to the greater overlapping of the heart by the lung), and then expands a little, but not to its original extent, this being due to disappearance of the lung reflex and persistence of the cardiac reflex. Examination by the fluorescent screen bears out this interpretation. It is obvious that this method of examination—by the fluorescent screen, and rubbing the skin of the precordial region —serves to differentiate a dilated heart from a pericardial effusion. Abrams further suggests that the real factor involved in balneo- and mechanical-therapeutics of dilated hearts is not the baths and exercises as such, but the cutaneous irritation provoked by these manœuvres; and he employs vigorous cutaneous friction by means of hand-rubbers, after immersing the patient in a warm bath for fifteen minutes to increase the sensitiveness of the skin. By this simple and expeditious method, in chronic heart disease, he can produce results emulating the conventional Schott method. Relief of dyspnœa follows, there is reduction in cardiac volume, besides a marked reduction in pulse rate with increase of volume and force.

**The Diagnosis of Thoracic and Cardiac Aueurysm by the Roentgen Rays** is described in a paper by Walsham,[5] who says that it has become the duty of a physician to make use of this method of examination in all doubtful cases of chest disease, especially if from the signs and symptoms an aneurysm be suspected. After detailing the methods of taking a radiograph, he mentions that frequently a small shadow is seen projecting to the left of the aortic arch which might easily be mistaken for an aneurysm—it is probably a normal shadow of the junction of the transverse with the descending thoracic aorta; it becomes accentuated if the tube be placed a little obliquely to the plate. For the diagnosis of an aneurysm the shadow must be in continuity with the heart or aorta. The shadow cast by an interthoracic new growth is quite different. The difference between the density of the heart shadow and the shadow of the aneurysmal sac is well marked, the latter being much the denser of the two. Since fluid blood is almost completely transparent to the rays, the greater the density of the shadow thrown by the aneurysmal sac the more abundant must be the laminated clot in the sac, for clotted blood is comparatively opaque to the rays. The position of the heart is an important sign of aortic aneurysm. For instance, a man, aged 51, complained of pain in the back between the shoulders and dyspnœa of three months' duration. He had well-marked signs of aortic regurgitation. There was no pulsation to be felt on the chest wall, no dysphagia, no cough, no cord paralysis, no definite tracheal tugging. The apex beat was displaced outwards, and on percussion the heart was found lying almost transversely in the chest. Thus, excepting for the position of the heart, the case might have been thought one of simple aortic regurgitation. On a screen examination, however, the patient was found to have an aneurysm of the aorta.

A case of **Gonorrheal Ulcerative Endocarditis** has been recorded by Lartigan,[6] who remarks that very few cases of purely gonococcal endocarditis have hitherto been described. The patient, who had had a urethral discharge for eight weeks previously, fell ill with septicæmic symptoms, an apical systolic murmur, and, later, arthritis of the right elbow joint. Post-mortem examination showed an ulcerative endocarditis of the mitral valve, infarction of the spleen, and cloudy swelling of the liver and kidneys. Bacteriological examination revealed the gonococcus in the heart's blood and valvular vegetations, with the bacillus coli communis in the liver, gall, and urinary bladders. Gonococcal urethritis may be the starting-point of a fatal septicæmia induced by a pure infection with the gonococcus, and endocarditis and arthritis are occasionally complications of such an infectious disease —as in the above described case—which, further, shows that the endocardial processes may be incited by the gonococcus without the association of other organisms. This complicating endocarditis of gonorrhœa is distinguished by no special anatomical feature from that induced by the more common pyogenic bacteria, and the symptomatology is essentially the same as that met with in the other forms of ulcerative lesion. Arthritis, when present, often precedes, less often follows, the cardiac affection.

**Aneurysm of the Coronary Arteries of the Heart.**—Griffith[7] has recorded two instances of aneurysm of the coronary arteries of the heart and collected the cases found in medical literature. In half the number death resulted from rupture into the pericardial sac. The aneurysms, which varied in size from that of a small pea to as large as a chestnut, were as a rule on the main stem of the artery or on the ventricular part of the heart. The causation of the aneurysm was in many cases embolism, as is so often the case in aneurysm of the smaller arteries of the body. The embolus is often derived from endocarditis of an infective character. The vessel dilates at the point of impact, and the softening of the vessel wall is due to the fact that the embolus is infective.

**The Treatment of Profuse Epistaxis in People beyond Middle Age.**—Coates[8] considers that the sequence of events that lead up to the epistaxis is as follows: (*a*) long-continued high arterial pressure; (*b*) some sudden cardiac failure; (*c*) overfilling of the whole venous system, the weakened heart not being able sufficiently to empty the engorged veins against the high pressure in the arterial system, due to contracted arterioles, and (*d*) leakage from an overfilled vein. The treatment should be directed to the small arteries and not to the nose. It is found that by giving nitroglycerine the general blood-pressure is lowered and the epistaxis ceases.

[1] Practioner, 1901. [2] Lancet, June 8, 1901. [3] Lancet, May 18, 1901. [4] Med. Record, January, 25, 1901. [5] Edin. Med. Jour., April, 1901. [6] Amer. Jour. of Med. Sc., January, 1901. [7] Brit. Med. Jour., February, 2, 1901. [8] Lancet, April 20, 1901.

